# Allostatic Load Model Associated with Indoor Environmental Quality and Sick Building Syndrome among Office Workers

**DOI:** 10.1371/journal.pone.0095791

**Published:** 2014-04-23

**Authors:** Chien-Cheng Jung, Hsiu-Hao Liang, Hui-Ling Lee, Nai-Yun Hsu, Huey-Jen Su

**Affiliations:** 1 Department of Environmental and Occupational Health, College of Medicine, National Cheng Kung University, Tainan city, Taiwan; 2 Department of Chemistry, College of Science and Engineering, Fu Jen Catholic University, New Taipei city, Taiwan; Tsinghua University, China

## Abstract

This study investigates whether indoor environmental quality (IEQ) influences allostatic load (AL) and whether AL can be a predictor for sick building syndrome (SBS). We also assessed and compared the associations between AL and SBS versus 8-hydroxydeoxyguanosine (8-OHdG) and SBS. A total of 115 office workers from 21 offices completed self-reported SBS questionnaires, and provided 11 biomarkers for their AL. Multiple linear regressions and logistic regression analysis were applied to examine the correlations between IEQ and AL or 8-OHdG and between AL or 8-OHdG and SBS, respectively. Our data revealed that the neuroendocrine system was correlated with CO_2_, the difference between indoor and outdoor CO_2_ levels (dCO_2_), and the indoor-outdoor ratio of CO_2_ (CO_2_ I/O). Metabolic system effects were associated with illumination. The relationships between illumination, CO_2_, dCO_2_, CO_2_ I/O and 8-OHdG were consistent with those and AL in specific systems. Furthermore, we found that risks for SBS syndromes were related with neuroendocrine and metabolic system of the AL. 8-OHdG was associated with eye dryness or irritation, eye tiredness and vomiting. We conclude that IEQ significantly influences AL and that AL can be a predictor for reporting SBS with information on system-specific effects.

## Introduction

Sick building syndrome (SBS) refers to situations in which building occupants suffer from discomforts of the nose, skin or eyes, and the symptoms are relieved when the occupants leave the building. Both personal and environmental factors may be determinants for SBS [1]. Environmental factors include ventilation rate, levels of total volatile organic compounds (TVOCs) or water dampness, etc. [2]–[5], while personal factors include stress from job or socioeconomic status, life quality, job satisfaction, and others [6]–[10].

Currently, researchers have investigated biomarkers that may be associated with SBS, one of which is 8-OHdG. The oxidized nucleoside 8-OHdG is the most frequently detected DNA lesion resulting from the action of reactive oxygen species, and upon DNA repair, this molecule is excreted in the urine [11]–[13]. Previous studies have found that urinary 8-OHdG in smokers and subjects of frequent exposure to traffic pollution is higher than that of non-smokers and subjects with low exposure to traffic pollution [14], [15]. Therefore, 8-OHdG can be adopted as an effect marker for exposure to pollution. Furthermore, urinary 8-OHdG is associated with both indoor pollutant exposure and self-reported SBS [16], [17]. Although these studies suggest that 8-OHdG is a potential marker for reporting SBS, the etiology of SBS has yet to be fully understood, and a comprehensive approach for examining SBS is not available.

Allostasis is a process that reestablishes homeostasis after the human body experiences neuroendocrine stress response [18]. However, continued or chronic stress can result in overactive stress responses or exhaustion, leading to wear and tear on the body, referred to as allostatic load (AL). In other words, frequent activation of stress response can damage the body over the long run and increase risks of cardiovascular diseases, diabetes, depression, or neurodegenerative diseases [19]–[21]. The occurrence of AL is often associated with stress from work, socioeconomic status [22]–[27], adverse childhood experiences, or chronic stress in childhood [28]–[31]. Given that AL is a physiological consequence of stress and that stress is one risk factor for SBS, understanding the relationship between SBS and AL may provide further insights for the etiology of SBS.

Recent studies indicate that environmental factors may derive various levels of biomarkers or physiologic parameters that are adopted to quantify AL. Systolic and diastolic pressure, heart rate, triacylglycerol, and hemoglobin A1c were found to be significantly increased upon exposure to increased PM_2.5_ and PM_10_ [32], [33]. A1c level was also significantly associated with ozone exposure [32]. In addition, exposure to TVOC, fungi or β-1,3-D Glucan caused elevated interleukin (IL)-6, tumor necrosis factor (TNF)-α and C-reactive protein (CRP) levels [34]–[37]. Perry and co-workers found that illumination was associated with creatinine (Perry et al., 1979). These studies suggest that AL could be affected by environmental factors, although there has been no investigation for whether environmental factors influence AL.

Our hypothesis is that indoor environmental quality may affect AL level. We therefore aimed to investigate the association between AL and indoor environmental quality in office workers and their corresponding office spaces, respectively. We further hypothesized that AL may account for the reporting of SBS symptoms, and therefore the relationship between AL and SBS symptoms was assessed. In view of the existing literature, we also measured 8-OHdG levels and investigated the relationships among 8-OHdG, IEQ and SBS, respectively.

## Materials and Methods

### Study design and study subjects

A cross-sectional study was carried out from July 2011 to December 2012. We randomly selected four organizations in Kaohsiung and Tainan, Taiwan, who agreed to participate in the study. Twenty-one office spaces and 143 office workers were randomly chosen from the four organizations mentioned above, and subjects younger than 20 years, part-time employees, and subjects with mental diseases were excluded from further analyses [38], [39]. Another 28 subjects were further eliminated either for the lack of their urine and saliva samples, or for missing information on the questionnaires. A total of 115 subjects were included in the final assessment. Indoor and outdoor environmental quality of these offices was assessed during office hours (09:00–17:00). Physiological measurements, urine and saliva samples were collected from participants at the end of the workday. All participants were also asked to complete a self-reported questionnaire including questions regarding their personal information and assessment of SBS. The study was approved (ER-99-040) by Institutional Review Board of National Cheng Kung University Hospital, Tainan, Taiwan. All participants signed and provided their consent form to study group.

### Indoor and outdoor environmental quality measurement

Indoor and outdoor levels of carbon monoxide (CO), carbon dioxide (CO_2_), total volatile organic compounds (TVOC), particulate matter diameter below 2.5 micrometers (PM_2.5_), fungi, bacteria and illumination were monitored simultaneously during office hours. CO and CO_2_ were examined by the Q-TRAK Indoor Air Quality Monitor (Model 7575, TSI Corporation, Shoreview, USA). A real-time RAE/PGM-730 instrument was used to measure the levels of TVOC (Model ppbRAE 3000, ProRAE Corporation, San Jose, USA). The level of PM_2.5_ was determined by a DUST-TRAK Aerosol Monitor (Model 8520, TSI Corporation, Shoreview, USA). Fungi and bacteria were sampled every three hours by a signal-stage Anderson bioaerosol sampler with the flow rate of 28.3 l/min and sampling time of one min. Concentrations of bacteria and fungi in the air were obtained by two culturing media and conditions: Dichloran Glycerol (DG-18) at 25±1°C for five days and Tryptic Soy Agar (TSA) at 30±2°C for two days. Illumination was measured by an Illumination instrument (CL2000, Minolta Co., Japan), and these data were measured on each subject' desk.

### Biomarkers and the measure

The AL model based on multi-systemic physiological measurements was used to estimate the AL [40]. Each participant received eleven non-invasive measurements categorized in five systems: (1) cardiovascular and respiratory system, including systolic blood pressure (SBP), diastolic blood pressure (DBP) and heart rate (Terumo, Japan); (2) anthropometric system, including body mass index (BMI) and body fat (OMRON, Japan); (3) neuroendocrine system, including epinephrine and norepinephrine in urine (liquid chromatography with tandem mass spectrometry, LC-MS/MS), and cortisol (enzyme-linked immunosorbent assay (ELISA), BD Biosciences, USA); (4) metabolic system, including creatinine in urine (liquid chromatography); and (5) immune system, including IL-6 and TNF-α in saliva (ELISA kit, BD Biosciences, USA). Epinephrine, norepinephrine and cortisol were expressed as micrograms per gram creatinine. Saliva and urine samples were centrifuged at 3000 rpm for 10 min at 4°C, and the supernatant was drawn and stored at −80°C until analysis. To stabilize epinephrine and norepinephrine in urine, 0.5 ml H_2_SO_4_ (6 N) was added to urine samples before storage. A simple count score was used as a measure of AL. The cut-off point was set to the 75^th^ percentile, and the number of biomarkers for which the individual falls into the highest risk quartile of each measure's sample distribution was taken as the AL score. Urinary 8-OHdG was determined by liquid chromatography with tandem mass spectrometry (LC-MS/MS).

### Questionnaire

The questionnaire contained questions for personal information and an SBS (SBS standard questionnaire, [41]). Queries for personal information included age, gender, working years, weekly working hours, education, monthly income, marital status, smoking behavior and exercise frequency. For SBS symptoms, participants self-assessed if during the past four weeks inside the building they had experienced any of the following conditions: eye dryness or irritation; stridor; headache; dry throat; tiredness; chest discomfort; nose itch; cough; eye tiredness; nervous feeling; pain in neck, shoulders, or back; sneeze; concentration difficulty; dizziness; depression; breathing difficulty; vomit; or skin dryness or itch.

### Statistical analysis

Chi-square was used to test the difference of SBS prevalence between males and females and the difference of personal characteristics. A multiple linear regression model was applied to investigate the association between indoor environmental quality and AL score or 8-OHdG level, and a logistic regression model was used to calculate odd ratio (OR). Data analysis was performed by SAS 9.2 software (SAS Institute Inc, Cary, USA). The statistical significance was set to *p*<0.05.

## Results

### Study population characteristics

The study recruited 115 office workers from 21 offices to assess the relationship between indoor environmental quality, allostatic load and SBS risk. Characteristics of the subjects are summarized in Table 1. Of the study population, 83 were male and 32 female, and the mean age was 34.2 years (SD = 5.7). The majority of participants (86.1%) were non-smokers, and approximately half (51.4%) habitually exercised. Other parameters are shown in Table 1.

**Table 1:** Summary statistics for characteristics of subjects.

Variables	Mean ±S.D. or n (%)
Age (years)	34.2±5.7
Males	83 (72.2%)
Working years	4.4±4.3
Weekly working hours	42.5±14.2
Education (years)	
≤12 (High school)	3 (2.6%)
13–16 (College)	66 (57.4%)
≥17 (Master or higher)	46 (40.0%)
Monthly income (NTD)*	
<20,000	2 (0.9%)
20,000–40,000	36 (31.3%)
40,000–60,000	28 (24.3%)
60,000–80,000	29 (25.2%)
80,000–100,000	8 (7.0%)
≥100,000	12 (10.4%)
Marital status (n, %)	
Unmarried or single	59 (51.3%)
Married or live together	54 (47.0%)
Others	2 (1.7%)
Smoking behavior (n, %)	
Non-smoker	99 (86.1%)
Smoker	16 (13.9%)
Exercise frequency (≥45 min per week. n, %)**	
Always	5 (4.5%)
Usually	
Often	16 (14.5%)
Sometime	57 (51.4%)
Never	11 (9.9%)

S.D. Standard deviation NTD: New Taiwanese Dollars.

*Missing 1 samples **Missing 4 samples.

Based on the allostatic load model proposed by Juster et al., we took multi-parameter measurements from 115 participants, and defined the AL score for each individual as the number of measures that fell outside of normal range [40]. The cut-off point was set to be the 75^th^ percentile of each measure's sample distribution in our study, as shown in Table 2. From a total of 11 biomarker measurements, the average total AL score was 2.6 (SD = 1.7) (Table 3), with the highest 7 and the lowest 0.

**Table 2:** Cutoff for each biomarker of allostatic load.

Biomarkers	Cutoff point
BMI (kg/m^2^)	≥26.8
Fat (%)	≥29.4
SBP (mmHg)	≥132.5
DBP (mmHg)	≥86.5
Heart rate (times/min)	≥83.0
IL-6 (pg/ml)	≥15.4
TNF-α (pg/ml)	≥10.7
Cortisol (µg/g creatinine)	≥20.8
Epinephrine (µg/g creatinine)	≥17.7
Norepinephrine (µg/g creatinine)	≥42.9
Creatinine (mg/dl)	≥109.3

**Table 3:** AL score and 8-OHdG level.

Biomarkers	Mean ±S.D.	Median	Min-Max
AL scores			
Cardio. and Resp. system	0.8±0.9	0	0–3
Anthropometric system	0.5±0.8	0	0–2
Neuroendocrine system	0.7±0.8	1	0–3
Immune system	0.4±0.7	0	0–2
Metabolic system	0.3±0.4	0	0–1
Total allostatic scores	2.6±1.7	2	0–7
8-OHdG (mg/g creatinine)	4.1±2.2	3.5	0.8–12.9

S.D. Standard deviation.

Cardio. and Resp. (Cardiovascular and respiratory).

All subjects were required to provide self-reported SBS symptoms according to a standard SBS questionnaire developed by the World Health Organization. We analyzed the prevalence of SBS symptoms, and found the most common symptom be eye tiredness while the least to be stridor (Table 4). In addition to eye tiredness, the 8 most common discomforts of more than 30% prevalence included eye dryness or irritation, nose itching, cough, nervous, pain in neck, shoulders or back, sneeze, concentration difficulty, and depression. We also discovered a generally higher prevalence rate of SBS in females than in males, and thus performed chi-square test to evaluate the significance of the observation. In support of this observation, several symptoms were significantly more prevalent (*p*<0.05) in women, including eye dryness or irritation, dry throat, eye tiredness, sneeze, concentration difficulty, dizziness, depression, vomit, and skin dryness or itch, as shown in Table 4.

**Table 4:** Prevalence of SBS in males and females.

Symptoms	All (%, n)	Male (%, n)	Female (%, n)	*p-value* *
Eye dryness or irritation	35.7 (41)	28.9 (24)	53.1 (17)	<0.05
Stridor	2.6 (3)	1.2 (1)	6.3 (2)	0.18
Headache	29.6 (34)	26.5 (22)	37.5 (12)	0.25
Dry throat	27.8 (32)	19.3 (16)	50.0 (16)	<0.05
Tiredness	27.8 (31)	26.5 (22)	28.1 (9)	0.81
Chest discomfort	13.9 (16)	10.8 (9)	21.9 (7)	0.14
Nose itch	31.2 (37)	27.7 (23)	43.8 (14)	0.12
Cough	36.5 (42)	33.7 (28)	43.8(14)	0.439
Eye tiredness	63.5 (73)	54.2 (45)	87.5 (28)	<0.05
Nervous feeling	33.9 (39)	30.1 (25)	43.8 (14)	0.19
Pain in neck, shoulders or back	55.7 (64)	50.6 (42)	68.8 (22)	0.10
Sneeze	38.3 (44)	28.9 (24)	62.5 (20)	<0.05
Concentration difficulty	45.2 (52)	38.6 (32)	62.5 (20)	<0.05
Dizziness	23.5 (27)	18.1 (15)	37.5 (12)	<0.05
Depression	33.0 (38)	24.1 (20)	56.3 (18)	<0.05
Breathing difficulty	12.2 (14)	8.4 (7)	21.9 (7)	0.06
Vomit	13.4 (15)	7.2 (6)	28.1 (9)	<0.05
Skin dryness or itch	26.1 (30)	18.1 (15)	46.9 (15)	<0.05

*Statistical significance at *p*<0.05.

### Indoor environmental quality

The indoor environmental indicators levels in the 21 offices are summarized in Table 5. The average PM_2.5_ level in our study (37.9±31.3 µg/m^3^) was higher than the Taiwan indoor air quality standards (35 µg/m^3^) while the average level of the other pollutants were all below standards for each substance. However, we detected an extraordinary TVOC level of 1130 ppb in one office, which was substantially higher than the standard of 560 ppb, and we found that this office was located next to a clean room for organic solvent usage. Furthermore, the highest PM_2.5_ and total fungi levels detected in this study of 143.7 µg/m^3^ and 3180.2 CFU/m^3^, respectively, were significantly greater than the standards of 35 µg/m^3^ and 1000 CFU/m^3^, respectively. Onsite investigation revealed that the office with high PM_2.5_ was naturally ventilated and located in the vicinity of roads with heavy traffic, and the indoor-to-outdoor ratio of PM_2.5_ was 0.80. On the other hand, the office with an extremely high level of fungi was located near areas of vegetation with natural ventilation, and the indoor-to-outdoor ratio of fungi was 0.90. Taken together, these findings indicate that types of ventilation and characteristics of external environment play an important role in indoor air quality.

**Table 5:** Indoor environmental quality in 21 office units.

Environmental indexes	Mean ±S.D.	Median	Range
Illumination (Lux)*	495.2±392.0	384.5	57.6–1485.0
CO (ppm)†	0.73±0.10	0.74	0.54–0.90
CO_2_ (ppm)†	742.0±212.3	664.2	464.0–1193.6
TVOC (ppb)†	539.1±348.8	380.6	1.4–1330.0
PM_2.5_ (µg/m^3^)	37.9±31.3	27.3	6.1–126.5
Total fungi (CFU/m^3^)	507.2±751.5	254.4	115.0–3180.2
Total bacterial (CFU/m^3^)	385.2±222.4	333.3	123.7–927.3
dCO_2_ (ppm)	285.5±190.3	264.2	31.2–701.9
CO_2_ I/O	1.62±0.38	1.65	1.07–2.43

S.D. Standard deviation.

*Illumination was measured from each subject's desk.

† CO, CO_2_ and TVOC missing 2, 2 and 1 samples, respectively.

### Relationship between indoor environmental quality and AL score

As shown in Table 6, the relationship between indoor environmental quality and AL was analyzed by a multiple linear regression model. Age, gender, smoking behavior, education attainment, monthly income, marital status and exercise frequency were found to can affect AL condition [23], [25]–[28], [30]. In this study, we adjusted these variables to avoid causing effects. We found no association between total AL score and any of the indoor environmental indicators in our study. However, the AL score on individual body systems was associated with specific environmental indexes. CO_2_, the concentration difference of indoor and outdoor CO_2_ (dCO_2_) and the ratio of indoor and outdoor CO_2_ (CO_2_ I/O ratio) levels which were a marker of ventilation rate, were associated with AL score on the neuroendocrine system (*p*<0.05). Furthermore, there was a significantly negative relationship between illumination and AL score on the metabolic system. These results suggest that indoor environmental quality influences homeostasis of the body, and each indicator may target specific body systems.

**Table 6:** β relating to indoor environmental quality, AL score and 8-OHdG level (Standard error).

Environmental factors	Total AL score	System AL score					8-OHdG
		Cardio. and Resp.	Anthropometric	Neuroendocrine	Immune	Metabolic	
Illumination (Lux)	0.000 (0.001)	0.000 (0.000)	0.000 (0.000)	0.000 (0.000)	−0.000 (0.000)	−0.000 (0.000)*	0.002 (0.000)*
CO_2_ (ppm)	0.001 (0.001)	0.001 (0.000)	−0.000 (0.000)	0.001 (0.001)*	−0.001 (0.000)	−0.000 (0.000)	0.000 (0.000)*
CO (ppm)	−0.515 (0.489)	−0.332 (0.248)	−0.065 (0.215)	−0.224 (0.234)	0.487 (0.210)	−0.124 (0.123)	−0.636 (0.536)
TVOC (ppm)	−0.000 (0.001)	0.000 (0.000)	−0.000 (0.000)	0.000 (0.000)	−0.000 (0.000)	−0.000 (0.000)	−0.000 (0.001)
PM_2.5_ (µg/m^3^)	−0.014 (0.006)	−0.002 (0.003)	−0.002 (0.003)	−0.006 (0.003)	−0.005 (0.003)	−0.002 (0.002)	0.006 (0.008)
Total bacteria (CFU/m^3^)	0.000 (0.001)	−0.001 (0.000)	0.001 (0.000)	0.000 (0.000)	−0.000 (0.000)	−0.000 (0.000)	0.002 (0.001)
Total fungi (CFU/m^3^)	−0.000 (0.000)	0.000 (0.000)	−0.000 (0.000)	−0.000 (0.000)	−0.000 (0.000)	−0.000 (0.000)	0.000 (0.000)
dCO_2_ (ppm)	0.001 (0.001)	0.001 (0.001)	−0.000 (0.000)	0.001 (0.001)*	−0.001 (0.001)	−0.000 (0.000)	0.004 (0.001)*
CO_2_ I/O ratio	0.619 (0.592)	0.348 (0.304)	0.019 (0.260)	0.561 (0.263)*	−0.229 (0.255)	−0.278 (0.145)	1.978 (0.621)*

Adjusted for age, gender, smoking behavior, education attainment, monthly income, marital status and exercise frequency.

*Statistical significance at *p*<0.05.

### Relationship between AL score and SBS

Logistic regression model was applied to calculate the odds ratio (OR) of SBS with AL score. The control variables included age, gender, smoking status, educational level, monthly income, marital status, exercise frequency, lux, CO, TVOC, PM_2.5_, bacteria, fungi CO_2_, dCO_2_ and CO_2_ I/O ratio, and results of the analysis were shown in Table 7. AL score on the neuroendocrine system was associated with reported cough and pain in neck, shoulders or back (*p*<0.05). AL scores on the metabolic system were significantly associated with depression (*p*<0.05). Collectively, our analysis suggests that the AL model could be a potential predictive marker for SBS.

**Table 7:** Odds ratio of SBS associated with AL scores and 8-OHdG level (Odds ratio 95% CI).

Symptoms	Total AL score	System AL score					8-OHdG
		Cardio. and Resp.	Anthropometric	Neuroendocrine	Immune	Metabolic	
Eye dryness or irritation	1.22(0.88–1.69)	1.26(0.66–2.38)	1.74(0.84–3.61)	1.28(0.62–2.66)	0.82(0.35–1.89)	1.00(0.25–4.02)	1.47(1.01–2.15)*
Headache	1.07(0.76–1.52)	1.03(0.52–2.03)	0.93(0.42–2.06)	1.05(0.48–2.31)	1.24(0.51–3.03)	1.43(0.31–6.51)	1.38(0.97–1.95)
Dry throat	1.25(0.90–1.74)	1.89(0.87–3.94)	1.77(0.82–3.83)	1.13(0.52–2.44)	1.12(0.48–2.63)	0.51(0.12–2.23)	1.38(0.97–1.97)
Tiredness	1.16(0.83–1.62)	1.38(0.70–2.74)	1.45(0.69–3.06)	1.02(0.47–2.20)	1.85(0.74–4.65)	0.20(0.04–1.01)	1.33(0.94–1.88)
Chest Discomfort	1.21(0.75–1.96)	1.06(0.41–2.77)	0.62(0.20–1.94)	1.95(0.73–5.23)	1.45(0.48–4.34)	4.84(0.47–50.30)	1.33(0.85–2.07)
Nose itch	0.82(0.58–1.16)	0.41(0.16–1.06)	0.57(0.24–1.32)	1.96(0.89–4.31)	0.68(0.25–1.84)	0.72(0.14–3.71)	1.39(0.97–1.97)
Cough	1.13(0.84–1.53)	0.97(0.51–1.85)	0.95(0.46–1.95)	3.31(1.36–8.06)**	0.97(0.46–2.08)	0.60(0.15–2.36)	1.27(0.91–1.76)
Eye tiredness	0.96(0.71–1.32)	1.18(0.62–2.26)	1.04(0.50–2.19)	1.20(0.59–2.43)	0.70(0.33–1.51)	0.29(0.07–1.28)	1.83(1.11–3.02)*
Nervousness	0.95(0.69–1.29)	0.74(0.36–1.51)	0.70(0.32–1.51)	1.02(0.50–2.08)	0.87(0.36–2.09)	3.34(0.81–13.84)	1.10(0.89–1.52)
Pain in neck, shoulders or back	1.13(0.85–1.49)	1.26(0.70–2.28)	0.96(0.51–1.81)	1.95(1.01–3.77)*	0.73(0.35–1.49)	0.93 (0.26–3.38)	1.07(0.79–1.46)
Sneeze	0.66(0.44–0.96)	0.34(0.13–0.91)	0.83(0.38–1.79)	1.22(0.55–2.73)	0.37(0.12–1.12)	0.18(0.03–1.05)	1.19(0.82–1.71)
Concentration difficulty	1.14(0.83–1.58)	1.15(0.59–2.23)	1.20(0.59–2.42)	1.76(0.85–3.62)	1.06 (0.48–2.32)	0.31(0.07–1.42)	1.45(0.96–2.19)
Dizziness	0.94(0.65–1.37)	0.57(0.24–1.36)	0.98(0.39–2.45)	1.39(0.57–3.41)	1.60(0.50–5.11)	0.64(0.13–3.32)	1.41(0.95–2.10)
Depression	1.16(0.82–1.64)	0.83 (0.41–1.67)	0.92(0.43–1.94)	1.41(0.66–3.01)	1.57(0.65–3.80)	6.15(1.29–29.39)*	1.24(0.86–1.80)
Breathing difficulty	0.86(0.46–1.61)	0.52(0.12–2.36)	0.76 (0.20–2.92)	2.57(0.61–10.80)	0.57(0.09–3.65)	–	1.49(0.74–2.98)
Vomit	0.90(0.59–1.37)	0.41(0.11–1.57)	0.36(0.10–1.24)	2.34(0.75–7.29)	1.38(0.45–4.23)	0.88(0.13–6.25)	1.91(1.11–3.27)*
Skin dryness or itch	1.36 (0.92–2.01)	0.43(0.75–3.97)	2.33 (0.90–6.06)	1.84(0.76–4.49)	0.64(0.22–1.86)	0.94(0.18–4.97)	1.01(0.71–1.45)

Adjusted for age, gender, smoking behavior, education attainment, monthly income, marital status, exercise frequency, lux, CO, CO_2_, TVOC, PM_2.5_, bacteria, fungi, CO_2_ indoor and outdoor ratio, different of indoor and outdoor CO_2._

*Statistical significance *p*<0.05 ** Statistical significance *p*<0.01.

### Relationships between 8-OHdG and indoor environmental quality and SBS

The average 8-OHdG level was 4.1±2.2 mg/g creatinine (table 4) and it was associated with illumination, CO_2_, dCO_2_ levels and CO_2_ I/O ratio (*p*<0.05) (table 6). Moreover, logistic regression model analysis shows that 8-OHdG level was correlated with likelihood of reporting eye dryness or irritation, eye tiredness and vomit (*p*<0.05) (table 7), which are part of the SBS.

## Discussion

The current study reveals that allostatic load can be influenced by indoor environmental quality in addition to previously proposed personal risk factors such as job stress, socioeconomic status, and hunger [25], [28], [29]. More remarkably, we also demonstrate for the first time that AL scores are associated with the risk of SBS. Based on these findings, we propose that AL may potentially serve as a predictive index for SBS, and compared to 8-OHdG as a general indicator for DNA lesions, the analysis of AL allows further investigation of specific physical systems to help determine major risk factors of SBS.

The study measured allostatic load by examining 11 physiologic parameters on 115 subjects. The average AL score obtained in this study is 2.6±1.7, which is close to previous studies, which ranged from 2.8 to 3.4 [23], [42]–[46]. Notably, in earlier studies blood samples were required for analysis of IL-6 and TNF-α, both of which were parameters for assessing the immunological system of AL [47], while our study measured these two parameters from the saliva samples. From the standpoint of feasibility of field assessment, saliva sample collection is non-invasive, easy to collect, and requires much lower cost than the collection and analysis of blood samples. More importantly, since they are produced upon systemic or local inflammation, IL-6 and TNF-α are secreted into both the circulatory system and the saliva, and salivary IL-6 and TNF-α are well correlated with plasma IL-6 and TNF-α, respectively [48]–[50]. In addition, recent studies found that immunological indexes in saliva can be used to predict effects of stress [51], [52]. Together, these studies suggest that saliva is a valid tool for assessing immunological system of AL, and that non-invasive sampling may be sufficient to estimate specific systems of AL.

Several biomarkers that compose the multi-system index for AL were shown to be influenced by air pollutants. For example, exposure to PM_2.5_ or PM_10_ can decrease lung function and increase blood pressure and heart rate in the cardiovascular and respiratory system [33], [53]–[55]. Exposure to volatile organic compounds was associated with the levels of IL-6 and TNF-α in the immune system [36], [37]. In addition, higher concentration of ozone exposure was associated with elevated glycated hemoglobin and blood pressure in the metabolic and cardiovascular and respiratory system, respectively [32]. Therefore, air pollution may be one risk factor for AL. The current study found that CO_2_ concentration, dCO_2_ and CO_2_ I/O ratio are associated with AL in the neuroendocrine system (*p*<0.05, Table 6). Additionally, the study found that intensity of illumination has a negative relationship with AL on the metabolic system (*p*<0.05, Table 6), consistent with an earlier study that serum creatine level is negatively related to light exposure and light intensity [56]. Together, our research has shown that air pollutants influence the levels of AL, and each pollutant may impact only specific bodily systems.

Sick building syndrome refers to discomforts resulted from synergistic or independent effects of personal and environmental factors, with symptoms including headache, dry throat and concentration difficulty [1]. Among 115 subjects in this study, the most common symptoms are eye tiredness (63.5%), pain in neck, shoulders or back (55.7%), and concentration difficulty (45.2%) (Table 4). Other symptoms of more than 30% prevalence include eye dryness, nose itch, cough, nervous, sneeze and depression, which are similar to those in previous studies [57], [58]. Moreover, our study reveals that female subjects are more prone than males to suffer from eye dryness or irritation, dry throat, eye tiredness, sneeze, concentration difficulty, dizziness, depression, vomit and skin dryness or itch. Consistently, earlier reports have indicated that female prevalence of eye irritation, tiredness, dryness, pain in throat, and skin dryness are higher than in males [57], [58]. These results imply that females may be more susceptible than males to SBS.

The focus of most previous studies on SBS was either on environmental risks or on personal factors, leaving synergistic effects unexplored and potential risk factors for SBS undiscovered. For example, work stress, one of the personal factors, not only influences the occurrence of AL [25], [28], [29], but also increases the risk of SBS [7]–[10]; but the relationship between AL and SBS has not been investigated previously. This study found that air pollutant affects the expression of AL, suggesting that AL is a combined effect of air pollutants and personal factors. More remarkably, our analysis indicates that AL on the neuroendocrine system is associated with cough and pain in the neck, shoulders or back (*p*<0.05, Table 7). Together with a previous reports finding that pressure levels represented by stress hormones were linked to the prevalence of SBS [7], [10], these data support the hypothesis that AL on the neuroendocrine system can influence the incidence of SBS. Our study identified that CO_2_, dCO_2_ and CO_2_ I/O ratio were positively associated with AL on the neuroendocrine system. Since high prevalence of SBS is known to be associated with low levels of indoor ventilation [2], [3], our results provide a possible mechanism that the neuroendocrine system mediates the effect of ventilation rate on the prevalence of SBS. In addition, the prevalence of depression is positively correlated with AL on the metabolic system in our study (*p*<0.05, Table 7). This is the first discovery of the relationship between depression and metabolic system and deserves further study in the future. Taken together, in the light of our research, AL appears to be a combinational result of air pollutants and personal factors, and AL score may be considered an assessment approach for identifying SBS.

Production of 8-OHdG is known to be induced by exposure to industrial pollutants such as benzene, engine oil or chromium [59]–[61], and its correlation with non-industrial pollutant exposure was firstly revealed by Lu and co-workers [16]. Subsequently, 8-OHdG was considered a likely predictive marker for SBS [17]. The current study further compares the feasibility and effectiveness of AL and 8-OHdG in predicting risk of reporting SBS, and analyzes the relationship between 8-OHdG, IEQ and SBS in the same group. The average concentration of 8-OHdG in our study was comparable with Lu's work, the SBS questionnaire was the standard WHO questionnaire, and some variables with personal and environmental factors were adjusted. Table 6 shows that selected indoor air quality indicators were associated with 8-OHdG level. The relationships between illumination, CO_2_, dCO_2_ and CO_2_ I/O ratio and 8-OHdG were consistent with those and AL in specific system. In particular, visible light is known to irradiate iron ion-binding molecules such as haem or myoglobin, generating reactive oxygen species OH., H_2_O_2_ or O_2_^−.^that in turn cause DNA damage and produce 8-OHdG [12], [62], [63]. Therefore, our findings of correlation analysis are consistent with previous mechanistic studies. Furthermore, our finding of an association between CO_2_ and 8-OHdG agrees with Lu's work, [16], yet we found no significant association between 8-OHdG and TVOC, as suggested in that work. It is possible that ppbRAE measures only total VOC levels and there are often different compositions of VOCs in different office spaces. Furthermore, we found that 8-OHdG was associated with reported eye dryness or irritation, eye tiredness and vomiting (table 7), and AL was also associated with SBS symptoms.

Different SBS symptoms are physiologically related to specific human body systems. AL, however, is profiled by summing outcomes of 5 individual body system and therefore can be assessed with different specific SBS. In this study, we found that the association between CO_2_ and “reported pain in the neck and lack of concentration” is explained, with biological plausibility, by the neuroendocrine system identified through the AL analysis. Furthermore, AL is shown to serve as surrogate outcomes responding to both environmental and personal stress [25], [27], [28], though no study to date has demonstrated any potential association between 8-OHdG levels and the characterization of personal factors. Therefore, AL may be considered far more attractive than 8-OHdG for appropriate application in research on the etiology of SBS.

Finally, we review a few limitations of this study. First, the sampling was carried out during working hours, and so the actual exposure to environmental factors could not be precisely quantified if the subjects were required to leave their seats for a substantial period of time due to increased work demands. Secondly, work stress was not exactly determined and thus is not included as an adjustable variable in our analysis. Furthermore, since only non-invasive sampling was used, there is no information on plasma C-reactive protein (CRP), total cholesterol (TC) and high-density lipoprotein (HDL) that could be used for further analysis of the association between AL and indoor air quality and SBS. Finally, although gender and exercise frequency were included as adjustable variables in assessing correlations, creatinine was the only marker used in our study to represent metabolic system of AL.

## Conclusions

AL is influenced by environmental pollutants and is positively associated with SBS, and compared to the single measure of 8-OHdG, multi-systemic AL investigation may provide significant insights for the etiology of SBS.
